# The influence of uterine fibroids on adverse outcomes in pregnant women: a meta-analysis

**DOI:** 10.1186/s12884-024-06545-5

**Published:** 2024-05-06

**Authors:** Hong Li, Zhonghua Hu, Yuyan Fan, Yingying Hao

**Affiliations:** 1 Department of Cardiology, Shengjing Hospital of China Medical University, Shenyang, Liaoning China; 2 Department of Obstetrics and Gynecology, Shengjing Hospital of China Medical University, No. 36 Sanhao Street, Heping District, Shenyang, 110004 China

**Keywords:** Uterine fibroids, Pregnancy, Obstetric outcome, Meta-analysis

## Abstract

**Objective:**

The objective of the meta-analysis was to determine the influence of uterine fibroids on adverse outcomes, with specific emphasis on multiple or large (≥ 5 cm in diameter) fibroids.

**Materials and methods:**

We searched PubMed, Embase, Web of Science, ClinicalTrials.gov, China National Knowledge Infrastructure (CNKI), and SinoMed databases for eligible studies that investigated the influence of uterine fibroids on adverse outcomes in pregnancy. The pooled risk ratio (RR) of the variables was estimated with fixed effect or random effect models.

**Results:**

Twenty-four studies with 237 509 participants were included. The pooled results showed that fibroids elevated the risk of adverse outcomes, including preterm birth, cesarean delivery, placenta previa, miscarriage, preterm premature rupture of membranes (PPROM), placental abruption, postpartum hemorrhage (PPH), fetal distress, malposition, intrauterine fetal death, low birth weight, breech presentation, and preeclampsia. However, after adjusting for the potential factors, negative effects were only seen for preterm birth, cesarean delivery, placenta previa, placental abruption, PPH, intrauterine fetal death, breech presentation, and preeclampsia. Subgroup analysis showed an association between larger fibroids and significantly elevated risks of breech presentation, PPH, and placenta previa in comparison with small fibroids. Multiple fibroids did not increase the risk of breech presentation, placental abruption, cesarean delivery, PPH, placenta previa, PPROM, preterm birth, and intrauterine growth restriction. Meta-regression analyses indicated that maternal age only affected the relationship between uterine fibroids and preterm birth, and BMI influenced the relationship between uterine fibroids and intrauterine fetal death. Other potential confounding factors had no impact on malposition, fetal distress, PPROM, miscarriage, placenta previa, placental abruption, and PPH.

**Conclusion:**

The presence of uterine fibroids poses increased risks of adverse pregnancy and obstetric outcomes. Fibroid size influenced the risk of breech presentation, PPH, and placenta previa, while fibroid numbers had no impact on the risk of these outcomes.

**Supplementary Information:**

The online version contains supplementary material available at 10.1186/s12884-024-06545-5.

## Introduction

Uterine fibroids are benign tumors that affect 1–10% of women of reproductive age [1–3]. As fibroids are usually asymptomatic, it is difficult to quantify the exact prevalence in the population. The prevalence in pregnant women, however, has been found to range from 1 to 10.7% [2–4] and as increasing numbers of women are delaying childbearing, these figures are likely to increase. Despite extensive investigation into ways of preventing and treating uterine fibroids, their underlying etiology is still unclear [5, 6].

Several studies have assessed the effects of uterine fibroids on pregnancy and obstetric outcomes. However, there are many inconsistencies in their findings on the relationships between fibroids and cesarean delivery, preterm delivery, breech presentation, placenta previa, preterm premature rupture of membrane (PPROM), postpartum hemorrhage (PPH), and intrauterine growth retardation (IUGR) [1, 4, 7–11] with some investigations suggesting associations between fibroids and these complications and others reporting no elevated risks linked to the presence of fibroids [12, 13]. The aim of this meta-analysis was to determine the influence of uterine fibroids on pregnancy and obstetric outcomes, specifically examining the effects of multiple or larger (≥ 5 cm in diameter) uterine fibroids on these adverse outcomes.

## Materials and methods

### Search strategy

This study was performed according to the Preferred Reporting Items for Systematic Reviews and Meta-Analyses (PRISMA) guidelines [14]. We searched several used electronic databases, including PubMed, Embase, Web of Science, ClinicalTrials.gov, China National Knowledge Infrastructure (CNKI), and SinoMed, from their incept to January 15, 2023. The search strategy details are presented in the supplementary file 1. The search was not restricted in terms of language or publication type. In addition, the reference lists of the included studies were manually searched to identify additional eligible articles that may have been omitted from the initial search.

### Study inclusion criteria

According to the prespecified protocol, all studies that examined the associations between uterine fibroids and pregnancy/obstetric outcomes in pregnant women were included. Eligible studies were randomized controlled trials (RCTs), cohort studies, case-control studies, or comparative trials, and had to provide the pregnancy/obstetric outcomes. Reviews, letters, case reports, editorials, and comments were not included. If a clinical trial had been published in several journals, only the most informative study or the study with the longest follow-up was included to prevent duplication.

### Data extraction

The extracted data included the following: (1) study information: name of first author, year of publication, country, sample size; (2) subjects’ information: sociodemographic and clinical characteristics, including maternal age, gestational age at delivery, gravidity, parity, body mass index (BMI), history of smoking, alcohol consumption, gestational diabetes mellitus, and hypertensive disorders; (3) outcome measures: cesarean delivery, fetal distress, breech presentation, intrauterine fetal death, IUGR, low birth weight, malposition, miscarriage, placenta previa, placental abruption, PPH, preeclampsia, preterm birth, and PPROM. Two independent investigators extracted the data and disagreements were addressed by discussion to reach a consensus.

### Quality assessment

Two independent investigators were responsible for the assessment of methodological quality. For RCTs, methodological quality was evaluated using the Risk of Bias 2.0 tool [15] while the Risk of Bias in Non-Randomized Studies of Interventions (ROBINS-I) was used for interventional non-RCTs [16]. For cross-sectional studies, the Newcastle-Ottawa (NOS) scale with specific adaptations was used [17].

### Statistical analysis

STATA software version 12.0 (Stata Corporation, College Station, TX, USA) was used for meta-analysis. Risk ratios (RRs) with 95% confidence intervals (95%CI) were used for dichotomous outcomes. Statistical heterogeneity among the included studies was assessed using the Cochrane Q and *I*^2^ statistics [18], in which *P* < 0.1 or *I*^2^ > 50% were considered to be significant. In the event of significant heterogeneity, a random-effects model [19] was used for pooling the estimate or a fixed-effects model [20] was used. Sensitivity analyses were used to determine the effect of single-trial exclusions on the overall estimate. Publication bias was assessed by Begg’s [21] and Egger’s [22] tests. A *P*-value less than 0.05 was considered statistically significant, except where otherwise specified.

### Subgroup analysis and data analysis after controlling for confounding factors

Subgroup analysis was performed to analyze the effects of uterine fibroid size (small [< 5 cm in diameter] vs. large [≥ 5 cm in diameter]) and number (single fibroids VS multiple fibroids). The size of leiomyomas was quantified by measuring the largest diameter. Consistent with previous research, we classified a fibroid as large when its diameter reached or exceeded 5 cm, as determined through ultrasonography [23].

Several of the included studies used univariate and multivariate logistic regression to assess relationships between uterine fibroids and pregnancy/obstetric outcomes. In these studies, the authors provided the adjusted odds ratio (OR) or RR after controlling for potential confounders, such as maternal age, race, BMI, parity, diabetes, hypertension, alcohol, and smoking. Thus, we extracted the adjusted values for data analysis.

### Meta-regression analysis

We hypothesized that various factors might have affected the results of the included studies; these included demographic (maternal age and BMI) and clinical (gravidity, parity, smoking status, diabetes mellitus, and hypertension) variables. We, therefore, conducted a meta-regression analysis to determine the possible effects of these variables on the reported results. In the regression model, the outcome was regarded as the dependent variable (y) and the covariates described above as the independent variables (χ).

## Results

### Study identification

**Fig. 1:**
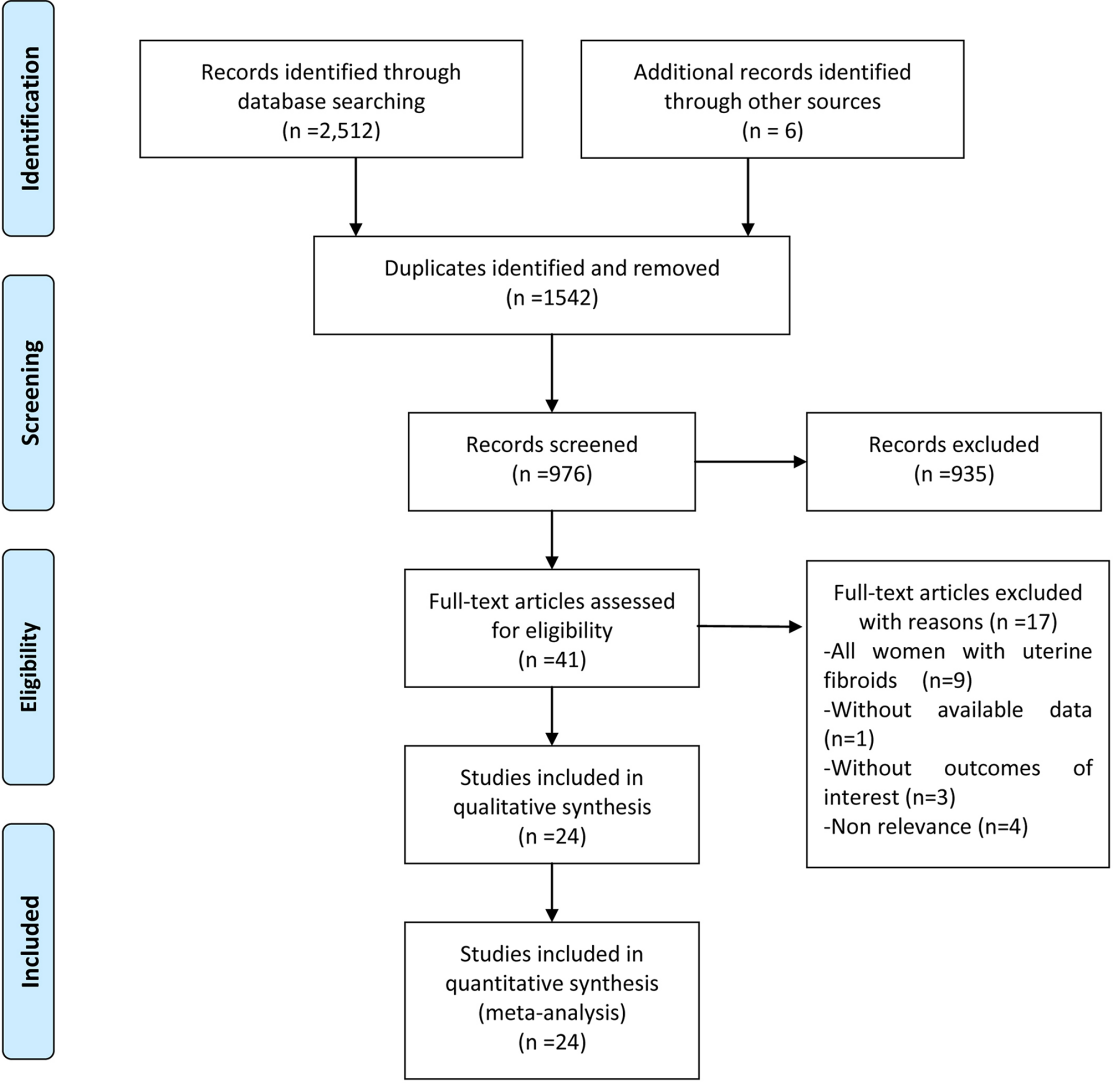
Eligibility of studies for inclusion in meta-analysis

A total of 2512 potentially relevant articles were identified from the database searches together with six additional articles from other sources. Of these, 1542 were duplicates and were removed, leaving 976 articles for article/abstract review. Of these, 935 were excluded because of various reasons. The full texts of the 41 remaining articles were reviewed, resulting in the exclusion of 17 articles. Finally, 24 studies [1, 4, 7, 12, 24–43] were considered to meet the inclusion criteria and were included for qualitative synthesis (Fig. 1).

### Characteristics of included studies and quality assessment

The baseline features of the included studies are shown in supplementary file 2. All the studies had a retrospective cohort design, with seventeen carried out in China [26, 28–43], five in the USA [1, 4, 7, 27], one in Italy [25], and one in France [24]. Sample sizes ranged from 127 to 112 403 participants. These studies included a total of 237 509 participants, of whom 10 560 were cases (women with uterine fibroids) and 226 949 were controls (women without uterine fibroids). As a consequence of family planning and the one-child policy, women from China tended to be primigravidae. In some of the included studies, women with uterine fibroids tended to be older, smokers, and drinkers, and to have higher BMI, or histories of diabetes mellitus and chronic hypertension, compared with those without fibroids.

Overall, the risk of bias in the cohort studies ranged from serious to low. Low bias risk was associated with intervention classification and analysis, deviations from intended interventions, and missing data. The risk of bias in confounding was deemed serious in four studies, with no information in three studies, critical in two studies, and low in the other studies. Bias risk in participant selection was found to be moderate in three studies, with no information in one study, and low in the other studies. Bias risk outcome measurement was moderate in one study and low in other studies while the risk in the selection of result reporting was deemed serious in two studies, moderate in three studies, and low in other studies. Overall, the risk of bias was critical in two studies [36, 37], serious in six studies [35, 38, 39, 41–43], moderate in five studies [27, 28, 30, 31, 33], and low in eleven studies [1, 4, 7, 12, 24–26, 29, 32, 34, 40] (Supplementary file 3).

### Preterm birth

Twenty-one studies [1, 4, 7, 12, 25–29, 31–33, 35–43] reported data on preterm birth. The preterm birth rate for pregnant women with uterine fibroids was 12.85% compared with 9.43% for the no-fibroid group. Pooled data showed that the presence of uterine fibroids posed a higher risk for preterm birth (RR = 1.72, 95%CI: 1.41, 2.10; *P* < 0.001). Significant heterogeneity was observed in the included studies (I^2^ = 74.5%, *P* < 0.001). Sensitivity analysis was conducted by the exclusion of an outlier trial [29] resulting in a slight alteration in the overall estimate (RR = 1.82, 95%CI: 1.49, 2.22; *P* < 0.001) with the heterogeneity still present (I^2^ = 73.6%, *P* < 0.001). Sensitivity analysis using the exclusion of a trial with a small sample size [39] also resulted in a small alteration in the pooled data (RR = 1.69, 95%CI: 1.39, 2.05; *P* < 0.001), with the heterogeneity remaining (I^2^ = 74.8%, *P* < 0.001). Further successive exclusion of the remaining single studies did not change the overall estimates and heterogeneity (data not shown).

### Cesarean delivery

Eighteen studies [1, 4, 7, 24–26, 29–31, 33, 35–40, 42, 43] reported data on cesarean delivery. The cesarean delivery rate in the fibroid group was 60.72% compared with 39.03% for the no-fibroid group. The aggregated data showed that fibroid presence led to an elevated risk of cesarean delivery (RR = 1.95, 95%CI: 1.67, 2.28; *P* < 0.001). The test for heterogeneity was significant (I^2^ = 96.8%, *P* < 0.001). The exclusion of an outlying trial [40] resulted in a slight change in the overall estimate slightly (RR = 1.86, 95%CI: 1.59, 2.17; *P* < 0.001) while heterogeneity was still present (I^2^ = 96.7%, *P* < 0.001). The exclusion of a trial with a small sample size [39] produced a similar effect, with no alteration in the overall estimate (RR = 1.92, 95%CI: 1.64, 2.25; *P* < 0.001) and the continued presence of heterogeneity (I^2^ = 96.9%, *P* < 0.001).

### Placenta previa

Sixteen studies [1, 4, 7, 24–26, 28, 29, 31, 32, 34, 38–42] reported data on placenta previa. The placenta previa rate for pregnant women with uterine fibroids was 2.48% compared with 0.98% for the no-fibroid group. The aggregated data showed that the presence of uterine fibroids significantly raised the risk of placenta previa (RR = 2.99, 95%CI: 2.06, 4.35; *P* < 0.001). There was significant heterogeneity (I^2^ = 65.6%, *P* < 0.001). The exclusion of an outlying trial [32] did not alter the overall estimate (RR = 2.86, 95%CI: 1.97, 4.15; *P* < 0.001) or the heterogeneity (I^2^ = 65.7%, *P* < 0.001). Similarly, the exclusion of a trial with a small sample size [39] did not affect the overall estimate (RR = 2.92, 95%CI: 1.99, 4.30; *P* < 0.001) or the heterogeneity (I^2^ = 67.1%, *P* < 0.001).

### Miscarriage

Fifteen studies [28–39, 41–43] reported data on miscarriage. The miscarriage rate for pregnant women with uterine fibroids was 13.42% compared with 2.84% for the no-fibroid group. The pooled data indicated a significantly elevated risk of miscarriage associated with the presence of fibroids (RR = 4.51, 95%CI: 2.80, 7.26; *P* < 0.001). Significant heterogeneity was observed (I^2^ = 51.3%, *P* = 0.011). The exclusion of an outlying trial [38] did not alter the overall estimate (RR = 4.28, 95%CI: 2.64, 6.94; *P* < 0.001) or heterogeneity (I^2^ = 51.8%, *P* = 0.012) significantly, while the exclusion of the trial with a small sample size [39] also did not affect the overall estimate (RR = 4.47, 95%CI: 2.70, 7.40; *P* < 0.001) or the heterogeneity (I^2^ = 54.3%, *P* = 0.008).

### Preterm premature rupture of membranes

Fifteen studies [1, 4, 7, 12, 24–27, 29, 35–37, 40, 42, 43] reported data on PPROM. The PPROM rates in the fibroid and no-fibroid groups were 9.65% and 9.53%, respectively. As shown by the pooled estimate, fibroid presence was associated with a significantly higher risk of PPROM in comparison with no fibroids (RR = 1.37, 95%CI: 1.09, 1.72; *P* < 0.001). Significant heterogeneity was observed (I^2^ = 74.7%, *P* < 0.001). However, sensitivity analysis involving the exclusion of single studies did not identify the source of the heterogeneity.

### Placental abruption

Fourteen studies [4, 12, 24, 27, 30, 35–37, 39, 43] reported data om placental abruption. The rate of placental abruption for pregnant women with uterine fibroids was 6.28% compared with 5.51% for the no-fibroid group. The pooled data indicated that fibroids significantly raised the risk of placental abruption (RR = 1.85, 95%CI: 1.48, 2.32; *P* < 0.001). No significant heterogeneity among the studies was observed (I^2^ = 36.3%, *P* = 0.086).

### Postpartum hemorrhage

Thirteen studies [26, 28–33, 35, 38, 40–42] reported data on PPH. The rate of PPH for pregnant women with uterine fibroids was 10.10% compared with 3.96% for the no-fibroid group. As shown by the pooled data, the presence of fibroids raised the risk of PPH significantly (RR = 3.52, 95%CI: 2.16, 5.73; *P* < 0.001). Significant heterogeneity was observed among the studies (I^2^ = 80.8%, *P* < 0.001) while sensitivity analysis was unable to identify the source of the heterogeneity.

### Fetal distress

Sixteen studies [28–43] reported data on fetal distress. The rates of fetal distress were 11.47% and 4.68% for the fibroid and no-fibroid groups, respectively and the pooled data indicated that the risk was significantly increased by the presence of fibroids (RR = 3.61, 95%CI: 2.08, 6.27; *P* < 0.001). Significant heterogeneity was observed (I^2^ = 72.8%, *P* < 0.001) while the source of the heterogeneity was not identified by sensitivity analysis.

### Malposition

Sixteen studies [28–43] reported data on malposition. The malposition rate for the fibroid group was 14.41% compared with 14.38% for the no-fibroid group with the pooled data showing a significant risk for malposition in the fibroid group (RR = 2.54, 95%CI: 1.75, 3.69; *P* < 0.001). Significant heterogeneity was observed (I^2^ = 64.7%, *P* < 0.001) which remained unaffected by sensitivity analysis.

### Intrauterine fetal death

Ten studies [1, 12, 29, 30, 33–37, 43] reported data on intrauterine fetal death. The rate of intrauterine fetal death in the fibroid group was 3.07% compared with 0.69% for the no-fibroid group, with pooled data showing a significant risk for intrauterine fetal death resulting from the presence of fibroids (RR = 2.34, 95%CI: 1.42, 3.84; *P* < 0.001). Significant heterogeneity was seen (I^2^ = 50.5%, *P* = 0.0033). Exclusion of an outlying trial [34] altered the overall estimate slightly (RR = 2.57, 95%CI: 1.95, 3.39; *P* < 0.001) and also eliminated the heterogeneity (I^2^ = 0.0%, *P* = 0.706), indicating that the trial of Xu JZ [34] was responsible for the heterogeneity among the included studies.

#### Low birth weight

Eight studies [12, 29, 31, 32, 34, 39, 40, 42] reported data on low birth weight. The rates for the fibroid and no-fibroid groups were 11.53% and 10.40%, respectively, with the pooled data showing a significant increase in the risk of low birth rate in the fibroid group (RR = 1.72, 95%CI: 1.03, 2.85; *P* < 0.001). Although significant heterogeneity was observed (I^2^ = 73.0%, *P* = 0.001), this remained unaffected by sensitivity analysis.

### Breech presentation

Six studies [1, 4, 7, 25, 26, 34] reported data on breech presentation. The rate of breech presentation for pregnant women with uterine fibroids was 8.30% compared with 3.70% for the no-fibroid group. Pooled data showed that the presence of uterine fibroids significantly elevated the risk of breech presentation (RR = 2.26, 95%CI: 1.56, 3.29; *P* < 0.001). Significant heterogeneity among the studies was observed (I^2^ = 91.2%, *P* < 0.001) but its source was not identified by sensitivity analysis.

#### Intrauterine growth retardation

Six studies [1, 25, 29, 31, 32, 34] reported data on IUGR. The rate of IUGR was 10.69% in the fibroid group compared with 12.97% in the no-fibroid group. The pooled data did not show any increased risk of IUGR associated with fibroids (RR = 1.25, 95%CI: 0.61, 2.55; *P* = 0.543). No significant heterogeneity was observed (I^2^ = 47.7%, *P* = 0.088).

#### Preeclampsia

Five studies [1, 4, 24, 29, 42] reported data on preeclampsia. The preeclampsia rates were 5.93% and 5.94% for the fibroid and no-fibroid groups, respectively, with pooled data indicating a significantly elevated risk in the fibroid group (RR = 1.48, 95%CI: 1.10, 2.00; *P* = 0.009). Significant heterogeneity among the studies was observed (I^2^ = 52.3%, *P* = 0.079).

#### Data analysis after adjusting for potential confounder factors

Nine studies [1, 4, 7, 12, 24, 26, 30, 33, 42] provided the adjusted values for controlling the potential confounder factors. The pooled data indicated that the presence of fibroids significantly elevated the risks of breech presentation (RR = 1.88, 95%CI: 1.18, 2.99; *P* = 0.008), placental abruption (RR = 1.94, 95%CI: 1.19, 3.16; *P* = 0.008), PPH (RR = 2.29, 95%CI: 1.78, 2.94; *P* < 0.001), preeclampsia (RR = 1.20, 95%CI: 1.02, 1.42; *P* = 0.031), intrauterine fetal death (RR = 1.82, 95%CI: 1.01, 3.28; *P* = 0.046), preterm birth (RR = 1.48, 95%CI: 1.12, 1.96; *P* = 0.006), cesarean delivery (RR = 2.13, 95%CI: 1.12, 4.04; *P* = 0.021), and placenta previa (RR = 1.62, 95%CI: 1.03, 2.53; *P* = 0.037). No significant associations were seen between fibroid presence and PPROM (RR = 1.30, 95%CI: 0.98, 1.72; *P* = 0.073) and low birth weight (RR = 1.36, 95%CI: 0.87, 2.13; *P* = 0.172) (Table 1).

**Table 1:** Results of meta-analysis: non-adjusted and adjusted analysis

Outcomes	Non-adjusted RR	*P*	Adjusted RR	*P*
Preterm birth	1.72 (1.41, 2.10)	**< 0.001**	1.48 (1.12, 1.96)	**0.006**
Cesarean delivery	1.95 (1.67, 2.28)	**< 0.001**	2.13 (1.12, 4.04)	**0.021**
Placenta previa	2.99 (2.06, 4.35)	**< 0.001**	1.62 (1.03, 2.53)	**0.037**
Miscarriage	4.51 (2.80, 7.26)	**< 0.001**	-	-
PPROM	1.37 (1.09, 1.72)	**< 0.001**	1.30 (0.98, 1.72)	0.073
Placental abruption	1.85 (1.48, 2.32)	**< 0.001**	1.94 (1.19, 3.16)	**0.008**
PPH	3.52 (2.16, 5.73)	**< 0.001**	2.29 (1.78, 2.94)	**< 0.001**
Fetal distress	3.61 (2.08, 6.27)	**< 0.001**	-	-
Malposition	2.54 (1.75, 3.69)	**< 0.001**	-	-
Intrauterine fetal death	2.34 (1.42, 3.84)	**< 0.001**	1.82 (1.01, 3.28)	**0.046**
Low birth weight	1.72 (1.03, 2.85)	**< 0.001**	1.36 (0.87, 2.13)	0.172
Breech presentation	2.26 (1.56, 3.29)	**< 0.001**	1.88 (1.18, 2.99)	**0.008**
IUGR	1.25 (0.61, 2.55)	0.543	-	-
Preeclampsia	1.48 (1.10, 2.00)	**0.009**	1.20 (1.02, 1.42)	**0.031**

*Abbreviation* PPROM, Preterm premature rupture of membranes; PPH, Postpartum hemorrhage; IUGR, Intrauterine growth retardation; RR, risk ratio

### Subgroup analysis of uterine fibroid size and number

Five studies [1, 7, 25–27] reported data that evaluated the effects of fibroid size and number on outcomes. The subgroup analysis analyzing fibroid size showed that the presence of large fibroids significantly elevated the risk of breech presentation (RR = 1.50, 95%CI: 1.03, 2.19; *P* = 0.036), placenta previa (RR = 5.04, 95%CI: 2.12, 12.01; *P* < 0.001), and PPH (RR = 1.62, 95%CI: 1.16, 2.25; *P* = 0.004), compared with small fibroids. Small fibroids, however, significantly raised the risk of breech presentation (RR = 1.40, 95%CI: 1.10, 1.79; *P* = 0.006), placental abruption (RR = 3.75, 95%CI: 2.83, 4.97; *P* < 0.001), cesarean delivery (RR = 1.48, 95%CI: 1.33, 1.65; *P* < 0.001), PPH (RR = 1.65, 95%CI: 1.41, 1.92; *P* < 0.001), and IUGR (RR = 1.15, 95%CI: 1.01, 1.30; *P* = 0.029), compared with an absence of fibroids (Table 2).

**Table 2:** Subgroup analysis based on the size of uterine fibroids and number of uterine fibroids

Outcomes	Large VS small fibroids		Small VS no fibroids		Multiple VS single fibroids	
	RR (95%CI)	*P*	RR (95%CI)	*P*	RR (95%CI)	*P*
Breech presentation	1.50 (1.03, 2.19)	**0.036**	1.40 (1.10, 1.79)	**0.006**	-	-
Placental abruption	0.84 (0.39, 1.78)	0.644	3.75 (2.83, 4.97)	**< 0.001**	1.22 (0.51, 2.94)	0.651
Cesarean delivery	0.98 (0.94, 1.02)	0.274	1.48 (1.33, 1.65)	**< 0.001**	0.85 (0.51, 1.50)	0.539
PPH	1.62 (1.16, 2.25)	**0.004**	1.65 (1.41, 1.92)	**< 0.001**	1.45 (0.53, 3.95)	0.464
Placenta previa	5.04 (2.12, 12.01)	**< 0.001**	0.95 (0.46, 1.97)	0.892	1.50 (0.90, 2.51)	0.122
PPROM	1.51 (0.88, 2.58)	0.132	1.04 (0.74, 1.46)	0.807	1.31 (0.55, 3.13)	0.545
Preterm birth	1.20 (0.89, 1.62)	0.227	1.67 (0.54, 5.12)	0.372	0.87 (0.50,1.44)	0.627
IUGR	0.90 (0.71, 1.15)	0.401	1.15 (1.01,1.30)	**0.029**	-	-

*Abbreviation* PPROM, Preterm premature rupture of membranes; PPH, Postpartum hemorrhage; IUGR, Intrauterine growth retardation; RR, risk ratio

Subgroup analysis of the effects of fibroid number showed that the presence of multiple fibroids did not increase the risk of PPROM (RR = 1.31, 95%CI: 0.55, 3.13; *P* = 0.545), placental abruption (RR = 1.22, 95%CI: 0.51, 2.94; *P* = 0.651), placenta previa (RR = 1.50, 95%CI: 0.90, 2.51; *P* = 0.122), preterm birth (RR = 0.87, 95%CI: 0.51, 1.50; *P* = 0.627), cesarean delivery (RR = 0.85, 95%CI: 0.50, 1.44; *P* = 0.539), and PPH (RR = 1.45, 95%CI: 0.53, 3.95; *P* = 0.464), compared with a single fibroid (Table 2).

### Meta-regression analysis

To further evaluate the influence of potential confounding factors (maternal age, BMI, parity, gravidity, smoking status, diabetes mellitus, and hypertension) on the outcomes, we conducted meta-regression analyses. While maternal age affected the difference in preterm birth between the fibroid and no-fibroid groups (t = 2.87, *P* = 0.012), other factors did not (Table 3). BMI influenced the difference in intrauterine fetal death (t = 3.13, *P* = 0.04) while other factors did not. None of these factors influenced malposition, fetal distress, PPROM, miscarriage, placenta previa, placental abruption, and PPH (Table 3).

**Table 3:** Meta-regression analysis for the impact of potential confounding factors

	Maternal Age			BMI			Gravidity			Parity		
	Coef(95%CI)	t	*P*	Coef (95%CI)	t	*P*	Coef (95%CI)	t	*P*	Coef (95%CI)	t	*P*
Preterm birth	21.05(5.34, 36.76)	2.87	**0.01**	5.31(-7.02, 17.65)	0.92	0.37	-9.69(-26.05, 6.68)	-1.27	0.23	-0.59(-15.90,14.72)	-0.08	0.94
Cesarean delivery	1.41(-0.36, 3.17)	1.74	0.11	0.35(-0.66, 1.36)	0.75	0.47	-0.34(-1.02, 1.34)	-0.44	0.67	-0.11(-1.59, 1.37)	-0.16	0.88
Malposition	15.01(-1.21,31.24)	2.06	0.07	3.30(-11.80,18.39)	0.49	0.64	-6.69(-13.82,0.45)	-2.09	**0.06**	-	-	-
Fetal distress	8.75(-2.89,20.38)	1.64	0.13	-	-	-	-3.96(-9.07,1.15)	-1.69	0.12	-	-	-
PPROM	-1.95(-8.84,4.95)	-0.65	0.53	0.59(-3.28,4.47)	0.35	0.73	-0.98(-7.14,5.18)	-0.37	0.72	3.34(-2.01,8.70)	1.44	0.19
Placenta previa	0.10(-5.96,6.17)	0.04	0.97	0.61(-3.89,5.11)	0.31	0.77	0.02(-5.97,6.02)	0.01	0.99	-0.74(-6.31,4.84)	-0.30	0.77
Placental abruption	4.42(-1.60,10.43)	1.66	0.13	1.53(-1.36,4.44)	1.20	0.26	-2.49(-7.69,2.72)	-1.08	0.31	0.18(-4.51,4.87)	0.09	0.93
PPH	36.25(-7.11,89.62)	1.61	0.15	2.19(-27.03,31.41)	0.18	0.86	-12.12(-29.0,4.77)	-1.70	0.13	-	-	-
Miscarriage	6.33(-2.55,15.20)	1.59	0.14	-	-	-	-3.22(-7.34,0.89)	-1.74	0.11	-	-	-
IFD	-0.67(-4.23,2.89)	-0.52	0.63	3.02(0.34,5.70)	3.13	**0.04**	-	-	-	0.76(-1.13,2.65)	1.12	0.33
	**Smoking**				**Diabetes**				**Hypertension**			
	**Coef (95%CI)**	**t**	***P***		**Coef (95%CI)**	**t**	***P***		**Coef (95%CI)**	**t**	***P***	
Preterm birth	4.27(-6.20, 14.75)	0.87	0.40		-3.48(-15.95, 8.99)	-0.60	0.56		5.22(-2.04, 12.48)	1.50	0.15	
Cesarean delivery	0.12(-0.93, 1.17)	0.25	0.81		-0.15(-1.29, 1.00)	-0.28	0.79		-0.13(-3.44, 3.17)	-0.09	0.93	
Malposition	-2.56(-15.54,10.41)	-0.44	0.67		0.97(-11.26,13.19)	0.18	0.86		-10.13(-54.77,34.50)	-0.51	0.62	
Fetal distress	-2.25(-10.77,6.27)	-0.58	0.57		1.55(-7.25.10.35)	0.38	0.71		-	-	-	
PPROM	-2.09(-5.67,1.50)	-1.34	0.22		-1.31(-4.95,2.34)	-0.83	0.43		-	-	-	
Placenta previa	2.96(-2.59,8.51)	1.20	0.26		-1.39(-7.72,4.94)	-0.5	0.63		-	-	-	
Placental abruption	-	-	-		-	-	-		-	-	-	
PPH	-5.49(-48.49,37.52)	-0.30	0.77		2.96(-36.59,42.53)	0.18	0.86		2.96(-36.60,42.53)	0.18	0.86	
Miscarriage	0.08(-6.62,6.78)	0.03	0.98		-0.09(-6.79,6.61)	-0.03	0.98		-	-	-	
IFD	-0.50(-2.96,1.96)	-0.57	0.60		-3.27(-5.58,0.96)	1.15	0.32		-	-	-	

*Abbreviation* PPROM, Preterm premature rupture of membranes; PPH, Postpartum hemorrhage; IFD, Intrauterine fetal demise; Coef, Coefficient; 95%CI: 95% confidence interval; BMI, body mass index

## Publication bias

The determination of potential publication bias using Egger’s and Begg’s tests indicated an absence of publication bias in the included studies (Egger’s test: t = 0.68, *P* = 0.533; Begg’s test: Z = 1.13, *P* = 0.260).

## Discussion

This meta-analysis was performed to investigate the impact of uterine fibroids on adverse pregnancy outcomes, specifically evaluating the effects of multiple or large (≥ 5 cm in diameter) uterine fibroids on the adverse outcomes. Our findings indicated that fibroids elevated the risk of certain pregnancy and obstetric outcomes, including preterm birth, cesarean delivery, placenta previa, miscarriage, PPROM, placental abruption, PPH, fetal distress, malposition, intrauterine fetal death, low birth weight, breech presentation, and preeclampsia. However, after adjustment, these negative effects were confined to preterm birth, cesarean delivery, placenta previa, placental abruption, PPH, intrauterine fetal demise, breech presentation, and preeclampsia.

Moreover, results from subgroup analysis showed a relationship between the presence of larger fibroids and significantly higher risks of breech presentation, PPH, and placenta previa compared with small fibroids. The presence of multiple fibroids did not increase the risk of breech presentation, placental abruption, cesarean delivery, PPH, placenta previa, PPROM, preterm birth, or IUGR.

In the meta-regression analysis, we also found that only maternal age affected the relationship between uterine fibroids and preterm birth, while BMI influenced the relationship between uterine fibroids and intrauterine fetal death. Other potential confounding factors had no impact on malposition, fetal distress, PPROM, miscarriage, placenta previa, placental abruption, and PPH.

The biological basis for the associations between fibroids and adverse outcomes is not clear. Several studies, however, have suggested that reduced uterine distension resulting from physical interference by the fibroids may be one of the reasons [44]. Moreover, women with fibroids have been found to have lower oxytocinase activity, leading to higher levels of oxytocin which, in turn, would lead to preterm contractions [45]. It is also possible that degraded submucosal fibroids may lead to chronic inflammation or infection, with the consequent production of cytokines potentially resulting in elevated risks for preterm delivery [13].

In this study, we screened the recent literature with the objective of evaluating the influence of uterine fibroids on adverse outcomes. Twenty-four studies were finally included in the analysis. All these studies used a retrospective cohort design and the ROBINS-1 method was, therefore, used to evaluate their quality. Eleven of the studies were considered to have a low risk of bias, with five showing moderate risk, six serious risk, and two showing a critical bias risk. Bias in cofounding factors and the selection of reported results can result in a low quality of evidence. Our research highlighted that several adverse outcomes were strongly associated with the presence of uterine fibroids. However, when we pooled data from studies that provided the adjusted RR estimate for confounding factors, we found that the risks of PPROM and low birth weight were not significantly raised by the presence of fibroids.

In the meta-regression, we noticed that maternal age was positively related to preterm birth (Coefficient = 21.05, *P* = 0.01). This finding agrees with previous evidence that the preterm birth risk increased with increasing maternal age [46–48]. Leader J, et al. [46] in a meta-analysis of 15 studies found that women with very advanced maternal ages (≥ 45 years old) had a 1.96-times greater likelihood of preterm birth. Similarly, in another meta-analysis [47] including 10 studies, the authors found that women of advanced maternal age (≥ 35 years old) were more likely to have preterm deliveries than younger women [35–40 years old (OR = 1.21, 95%CI: 1.16, 1.27) and those > 40 years old (OR = 1.18, 95%CI: 1.10, 1.27)]. Besides maternal age, the meta-regression found that BMI was significantly related to intrauterine fetal death. Aune D, et al. [49] in a systematic review and meta-analysis comprising 38 cohort studies investigating whether specific levels of BMI increased the likelihood of fetal or infant death found that the pooled RR per five-unit increase in BMI for fetal death was 1.21 (95%CI: 1.09, 1.35) and was 1.24 (95%CI: 1.18, 1.30) for stillbirth.

In this meta-analysis, we not only investigated the effects of uterine fibroids on major outcomes, such as preterm birth, cesarean delivery, breech presentation, malposition, fetal distress, PPROM, miscarriage, IUGR, placenta previa, and placental abruption but have also analyzed the outcomes in terms of fibroid size and number. Although several studies have investigated these aspects, the results were inconsistent due to small sample sizes. Here, we found that women with fibroids greater than 5 cm in diameter had an increased risk of breech presentation, PPH, or placenta previa, compared with women with fibroids less than 5 cm. However, in terms of fibroid numbers, we observed that the presence of multiple fibroids did not increase the risk of placental abruption, cesarean delivery, PPH, placenta previa, PPROM, and preterm birth, compared with single fibroids. These results suggested that only the size of the fibroids influenced the risk of breech presentation, PPH, and placenta previa, while fibroid numbers did not affect these outcomes. There are very few studies on the associations between fibroid size and number on adverse outcomes. Our results provide valuable information for the identification of the risks of breech presentation, PPH, and placenta previa.

This meta-analysis has several potential limitations. First, the meta-analysis pooled data from 24 studies with 237 509 participants; while this increased the statistical power for determining the influence of uterine fibroids on adverse outcomes, it also led to heterogeneity. Some heterogeneity might be explained by differences in geographical locations and participants’ characteristics. We conducted sensitivity analyses to identify the potential sources of this heterogeneity; unfortunately, this was unable to identify the sources. Second, several of the included studies did not adjust for potential confounding factors, and the factors selected for adjustment differed across the studies. This might have influenced the overall estimate. Third, the meta-analysis was based on retrospective cohort studies. Such studies are subject to selection bias as they rely on care utilization and imaging data and recruit subjects only from academic medical centers. Fourth, most of the included studies were conducted in China, which may prevent the broad generalizability of our results. Finally, it is important to note that due to the limitations of the available data, we were unable to conduct subgroup analyses based on fibroid size, specifically for those with a diameter exceeding 10 cm, and fibroid location. This limitation restricted our ability to fully explore the impact of these variables on the outcomes.

Despite these weaknesses, our meta-analysis has some notable strengths. First, the large sample size of 237 509 participants from 24 included studies resulted in increased statistical power and, consequently, more reliable and accurate findings. Second, our study provided more comprehensive information compared with previous meta-analyses. Other meta-analyses have only focused on one pregnancy or obstetric outcomes, such as placenta abruption, placenta previa, or preterm birth [50–52]. Third, to minimize the effect of confounding factors on our results, we corrected our analyses for multiple confounders using meta-regression analysis and pooled data. Fourth, we developed a complete and comprehensive search strategy, as well as accessing articles from multiple databases and the gray literature, to minimize missing potential studies. We also used stringent screening criteria in the literature selection and strict statistical methods in the data analysis to improve the accuracy of our results. Last, we included recently published studies, which ensures that our results are more applicable to present clinical settings.

In conclusion, this meta-analysis suggested that women with uterine fibroids have elevated risks of pregnancy and obstetric outcomes. Specifically, fibroid size was found to influence the risk of breech presentation, PPH, and placenta previa, while the number of fibroids did not affect the risk of these outcomes.

### Electronic supplementary material

Below is the link to the electronic supplementary material.

Supplementary Material 1

Supplementary Material 2

Supplementary Material 3

## Data Availability

The datasets generated and analyzed during the current study are available from the corresponding author on reasonable request.
