# Supplementary material for: The influence of uterine fibroids on adverse outcomes in pregnant women: a meta-analysis
Source: BMC Pregnancy Childbirth. 2024 May 6;24:345. doi: 10.1186/s12884-024-06545-5 (PMC11071265; doi:10.1186/s12884-024-06545-5)
Supplement: Supplementary file 1 — Supplementary Material 1 [file 12884_2024_6545_MOESM1_ESM.docx]

**Supplementary file 1: Literature search strategy**

**Pubmed**

1. "uterine fibroids"[MeSH Terms]
2. "Leiomyomas"[Title/Abstract] OR "fibroid tumor"[Title/Abstract] OR "fibroid tumors"[Title/Abstract] OR "tumor fibroid"[Title/Abstract] OR (("cysts"[MeSH Terms] OR "cysts"[All Fields] OR "cyst"[All Fields] OR "neurofibroma"[MeSH Terms] OR "neurofibroma"[All Fields] OR "neurofibromas"[All Fields] OR "tumor s"[All Fields] OR "tumoral"[All Fields] OR "tumorous"[All Fields] OR "tumour"[All Fields] OR "neoplasms"[MeSH Terms] OR "neoplasms"[All Fields] OR "Tumor"[All Fields] OR "tumour s"[All Fields] OR "tumoural"[All Fields] OR "tumourous"[All Fields] OR "tumours"[All Fields] OR "Tumors"[All Fields]) AND "Fibroid"[Title/Abstract]) OR "Fibromyoma"[Title/Abstract] OR "Fibromyomas"[Title/Abstract] OR "Fibroid"[Title/Abstract] OR "Fibroids"[Title/Abstract] OR "fibroid uterus"[Title/Abstract] OR "uterus fibroid"[Title/Abstract] OR (("Fibroma"[MeSH Terms] OR "Fibroma"[All Fields] OR "Fibromas"[All Fields]) AND "Uterine"[Title/Abstract]) OR "fibromas uterine"[Title/Abstract] OR "uterine fibroma"[Title/Abstract] OR "uterine fibromas"[Title/Abstract] OR "fibroids uterine"[Title/Abstract] OR "fibroid uterine"[Title/Abstract] OR "uterine fibroid"[Title/Abstract] OR "uterine fibroids"[Title/Abstract] OR "leiomyoma uterine"[Title/Abstract]
3. 1 OR 2
4. "Pregnancy Outcome"[MeSH Terms]
5. "pregnancy outcomes"[Title/Abstract] OR "outcome pregnancy"[Title/Abstract] OR "outcomes pregnancy"[Title/Abstract]
6. 4 OR 5
7. 3 AND 6

**Embase**

1. uterine fibroids/
2. (Leiomyomas* or Fibroid Tumor* or Fibroid Tumors* or Tumor, Fibroid or Tumors, Fibroid* or Fibromyoma* or Fibromyomas* or Fibroid* or Fibroid Uterus* or Uterus, Fibroid* or Fibroma, Uterine* or Uterine Fibrom*a or Fibroids, Uterine* or Uterine Fibroids or Leiomyoma, Uterine*).ti,ab.
3. 1 or 2
4. Pregnancy Outcome/
5. (Pregnancy Outcomes* or Outcome, Pregnancy* or Outcomes, Pregnancy*).ti,ab.
6. 4 or 5
7. 3 and 6

**Web of science**

1. TS=(Uterine fibroids or Leiomyomas or Fibroid Tumor or Fibroid Tumors or Tumor, Fibroid or Tumors, Fibroid or Fibromyoma or Fibromyomas or Fibroid or Fibroid Uterus or Uterus, Fibroid or Fibroma, Uterine or Uterine Fibroma or Fibroids, Uterine or Uterine Fibroids or Leiomyoma, Uterine)
2. TS=(Pregnancy Outcomes or Outcome, Pregnancy or Outcomes, Pregnancy)
3. #1 AND #2
