# Supplementary material for: The influence of uterine fibroids on adverse outcomes in pregnant women: a meta-analysis
Source: BMC Pregnancy Childbirth. 2024 May 6;24:345. doi: 10.1186/s12884-024-06545-5 (PMC11071265; doi:10.1186/s12884-024-06545-5)
Supplement: Supplementary file 2 — Supplementary Material 2 [file 12884_2024_6545_MOESM2_ESM.docx]

**Supplementary file 2**. Baseline characteristics of patients in the trials included in the meta-analysis

| Study | Country | Study design | Fibroids status | No. of patients | Age (mean ±SD, y) | Primipara |
| --- | --- | --- | --- | --- | --- | --- |
| Stout MJ[1] | USA | Retrospective cohort | Uterine fibroids | 2058 | 35.1±4.6 | NR |
|  |  |  | No fibroids | 61989 | 30.0±6.3 | NR |
| Coronado GD[4] | USA | Retrospective cohort | Uterine fibroids | 2065 | NR | 954 |
|  |  |  | No fibroids | 4243 | NR | 3268 |
| Qidwai GI[7] | USA | Retrospective cohort | Uterine fibroids | 401 | 33.7 | 230 |
|  |  |  | No fibroids | 14703 | 28.6 | 78596 |
| Lai J[12] | Portland | Retrospective cohort | Uterine fibroids | 401 | NR | NR |
|  |  |  | No fibroids | 14703 | NR | NR |
| Girault A[24] | France | Retrospective cohort | Uterine fibroids | 301 | 36.1±4.0 | 108 |
|  |  |  | No fibroids | 19565 | 32.0±5.4 | 8024 |
| Ciavattini A[25] | Italy | Retrospective cohort | Uterine fibroids | 219 | 34.8±4.2 | NR |
|  |  |  | No fibroids | 219 | 34.8±4.2 | NR |
| Zhao R[26] | China | Retrospective cohort | Uterine fibroids | 3012 | 32.0±4.9 | NR |
|  |  |  | No fibroids | 109391 | 27.9±5.2 | NR |
| Shavell VI[27] | USA | Retrospective cohort | Uterine fibroids | 95 | 32.2±5.5 | NR |
|  |  |  | No fibroids | 95 | 31.9±5.6 | NR |
| Xie HX[28] | China | Retrospective cohort | Uterine fibroids | 112 | 25-42 | 77 |
|  |  |  | No fibroids | 140 | 22-43 | 85 |
| Zhu LR[29] | China | Retrospective cohort | Uterine fibroids | 183 | 21-42 | 166 |
|  |  |  | No fibroids | 183 | 20-39 | 160 |
| Wang H[30] | China | Retrospective cohort | Uterine fibroids | 120 | 28.52±3.86 | 120 |
|  |  |  | No fibroids | 120 | 28.13±3.64 | 120 |
| Feng XP[31] | China | Retrospective cohort | Uterine fibroids | 132 | 24-44 | 53 |
|  |  |  | No fibroids | 144 | 22-45 | 59 |
| Han LQ[32] | China | Retrospective cohort | Uterine fibroids | 102 | 31.5±5.5 | 15 |
|  |  |  | No fibroids | 100 | 31.5±5.5 | 15 |
| Lv ZH[33] | China | Retrospective cohort | Uterine fibroids | 110 | 34.6±3.1 | NR |
|  |  |  | No fibroids | 112 | 34.5±3.2 | NR |
| Xu JZ[34] | China | Retrospective cohort | Uterine fibroids | 150 | 32.0±2.5 | 75 |
|  |  |  | No fibroids | 150 | 28.0±1.5 | 95 |
| Zhang Y[35] | China | Retrospective cohort | Uterine fibroids | 100 | 26.13±2.97 | 37 |
|  |  |  | No fibroids | 100 | 27.26±3.08 | 30 |
| Wu AP[36] | China | Retrospective cohort | Uterine fibroids | 100 | 31.6±4.2 | NR |
|  |  |  | No fibroids | 100 | 30.5±3.8 | NR |
| Wu CZ[37] | China | Retrospective cohort | Uterine fibroids | 70 | 29.0±2.5 | 7 |
|  |  |  | No fibroids | 70 | 28.8±2.6 | 5 |
| Zhou LN[38] | China | Retrospective cohort | Uterine fibroids | 132 | 24-44 | 53 |
|  |  |  | No fibroids | 144 | 22-45 | 59 |
| Wu LP[39] | China | Retrospective cohort | Uterine fibroids | 62 | 31.2±1.1 | 30 |
|  |  |  | No fibroids | 62 | 30.3±2.1 | 29 |
| Yang NN[40] | China | Retrospective cohort | Uterine fibroids | 302 | 21-44 | NR |
|  |  |  | No fibroids | 283 | 22-45 | NR |
| Wang LH[41] | China | Retrospective cohort | Uterine fibroids | 65 | 22-39 | 10 |
|  |  |  | No fibroids | 65 | 20-41 | 11 |
| Wang Y[42] | China | Retrospective cohort | Uterine fibroids | 168 | 30.0±2.5 | 18 |
|  |  |  | No fibroids | 168 | 28.5±2.4 | 13 |
| Xue HZ[43] | China | Retrospective cohort | Uterine fibroids | 100 | 30±6 | 22 |
|  |  |  | No fibroids | 100 | 29±5 | 20 |

**Abbreviation**: SD, standard deviation; NR, not reported; BMI, body mass index.
