# Supplementary material for: The influence of uterine fibroids on adverse outcomes in pregnant women: a meta-analysis
Source: BMC Pregnancy Childbirth. 2024 May 6;24:345. doi: 10.1186/s12884-024-06545-5 (PMC11071265; doi:10.1186/s12884-024-06545-5)
Supplement: Supplementary file 3 — Supplementary Material 3 [file 12884_2024_6545_MOESM3_ESM.docx]

**Supplementary file 3. Assessment of Risk of Bias in Non-randomized studies-of Interventions**

| **Study** | **Bias due to confounding overall** | **Bias in selection of participants into the study overall** | **Bias in classification of interventions overall** | **Bias due to deviations from intended interventions overall** | **Bias due to missing data overall** | **Bias in measurement of outcomes overall** | **Bias in selection of the reported result overall** | **Overall** |
| --- | --- | --- | --- | --- | --- | --- | --- | --- |
| Stout MJ[1] | Low | Low | Low | Low | Low | Low | Low | Low |
| Coronado GD[4] | Low | Low | Low | Low | Low | Low | Low | Low |
| Qidwai GI[7] | Low | Low | Low | Low | Low | Low | Low | Low |
| Lai J[12] | Low | Low | Low | Low | Low | Low | Low | Low |
| Girault A[24] | Low | Low | Low | Low | Low | Low | Low | Low |
| Ciavattini A[25] | Low | Low | Low | Low | Low | Low | Low | Low |
| Zhao R[26] | Low | Low | Low | Low | Low | Low | Low | Low |
| Shavell VI[27] | Low | Low | Low | Low | Low | Low | Moderate | Moderate |
| Xie HX[28] | No information | Moderate | Low | Low | Low | Low | Low | Moderate |
| Zhu LR[29] | Low | Low | Low | Low | Low | Low | Low | Low |
| Wang H[30] | No information | Moderate | Low | Low | Low | Moderate | Low | Moderate |
| Feng XP[31] | Low | Low | Low | Low | Low | Low | Moderate | Moderate |
| Han LQ[32] | Low | Low | Low | Low | Low | Low | Low | Low |
| Lv ZH[33] | No information | Low | Low | Low | Low | Low | Moderate | Moderate |
| Xu JZ[34] | Low | Low | Low | Low | Low | Low | Low | Low |
| Zhang Y[35] | Serious | Low | Low | Low | Low | Low | Low | Serious |
| Wu AP[36] | Critical | Low | Low | Low | Low | Low | Low | Critical |
| Wu CZ[37] | Critical | Low | Low | Low | Low | Low | Low | Critical |
| Zhou LN[38] | Low | Low | Low | Low | Low | Low | Serious | Serious |
| Wu LP[39] | Serious | Low | Low | Low | Low | Low | Low | Serious |
| Yang NN[40] | Low | Low | Low | Low | Low | Low | Low | Low |
| Wang LH[41] | Serious | Moderate | Low | Low | Low | Low | Low | Serious |
| Wang Y[42] | Low | Low | Low | Low | Low | Low | Serious | Serious |
| Xue HZ[43] | Serious | No information | Low | Low | Low | Low | Low | Serious |
